# Schistosomal Rectal Polyposis Mimicking Colorectal Cancer: A Case Report of a 60‐mm Polyp in a Young Ethiopian Man Treated at Firoomsis General Hospital

**DOI:** 10.1155/crgm/5589986

**Published:** 2026-09-28

**Authors:** Alemayehu Abebe Guji, Birhanu Gudeta Geleta, Gashahun Mekonnen Dissassa, Mohammed Merga Ayana, Melese Abere Gizaw, Kedir Negesso Tukeni

**Affiliations:** ^1^ Jimma University, Jimma, Ethiopia, ju.edu.et

**Keywords:** colorectal cancer mimic, colorectal polyp, endoscopy, Ethiopia, histopathology, Jimma, rectal polyposis, schistosomiasis

## Abstract

**Background:**

*Schistosomiasis* is a major public health problem in endemic regions, affecting over 250 million individuals globally, with *Schistosoma mansoni* frequently causing intestinal and hepatosplenic disease. Chronic infection may lead to rectal polyposis, which can mimic colorectal malignancy and create diagnostic and therapeutic challenges. Addressing these intersecting diagnostic and therapeutic issues necessitates a comprehensive multidisciplinary approach that integrates increased clinical vigilance in endemic populations, accurate histopathological assessment, focused endoscopic treatment, and antiparasitic therapy. This strategy aims to prevent patients from undergoing unnecessary and debilitating radical surgery.

**Case Presentation:**

We report a 25‐year‐old man from rural Ethiopia who presented with abdominal pain, hematochezia, anemia, and weight loss. Colonoscopy revealed a large pedunculated rectal polyp measuring 60 mm, along with multiple smaller sessile polyps. Histopathology confirmed *Schistosoma mansoni* ova with intense eosinophilic infiltration and no dysplasia. The patient was treated with praziquantel and iron supplementation; the smaller polyps were completely removed with cold snare polypectomy, and the largest polyp was removed by piecemeal resection with hot snare polypectomy.

**Outcome:**

At the 3‐month follow‐up, hemoglobin levels improved, stool microscopy was negative for *S. mansoni ova*, and colonoscopy demonstrated complete mucosal healing with no recurrent polyps.

**Conclusion:**

*Schistosomal* rectal polyposis closely resembles colorectal cancer, particularly in endemic regions. Histopathological and parasitological confirmation is essential to avoid unnecessary radical surgeries. Early diagnosis, appropriate antiparasitic therapy, and endoscopic management can achieve favorable outcomes, while follow‐up colonoscopy ensures long‐term disease control.

## 1. Introduction

*Schistosomiasis* is a waterborne parasitic disease caused by trematodes of the genus *Schistosoma*. Different species have distinct geographic distributions and organ predilections: *Schistosoma mansoni* is common in Africa, South America, and the Middle East and primarily causes intestinal and hepatic diseases; *S. haematobium* is associated with urogenital disease; and *S. japonicum* and *S. mekongi* affect the liver and intestines in Asia [1, 2]. Transmission occurs when infectious cercariae shed from freshwater snails penetrate human skin, migrate through the lungs and liver, and settle in the venous plexus of the mesenteric and perivesical circulatory systems. Adult parasites lay thousands of ova that trigger inflammation and fibrosis in host tissues [3].

Globally, *schistosomiasis* remains a neglected tropical disease with high morbidity rates. Acute *schistosomiasis* (Katayama fever) presents with fever, pruritus, and eosinophilia, whereas chronic *schistosomiasis* results from the host immune response to deposited eggs and leads to periportal fibrosis, portal hypertension, intestinal parasitosis, urogenital involvement, and glomerulonephritis [4]. In Ethiopia, more than 18 million people live in areas endemic to *S. mansoni,* with transmission driven by freshwater snail hosts such as *Biomphalaria* [5, 6]. This high burden translates to substantial gastrointestinal morbidity, including colitis, protein‐losing enteropathy, and polyposis.

Intestinal polyposis due to *schistosomiasis* is a common presentation in endemic regions but is rare in nonendemic areas [7–9]. Approximately 20% of infections in endemic settings result in intestinal polyposis [7, 8]. These polyps may enlarge, bleed, or obstruct, producing clinical features that mimic those of colorectal malignancy. Such overlap creates a diagnostic dilemma, particularly in resource‐limited settings, where advanced imaging and pathology services are scarce. Misdiagnosis can lead to unnecessary radical surgery, underscoring the importance of histopathological confirmation of diagnosis.

This case report from Ethiopia illustrates the clinical impact of *schistosomal* rectal polyposis, highlighting how a large 60‐mm polyp closely resembles colorectal cancer. By emphasizing the prevalence, diagnostic pitfalls, and management strategies, this report underscores the need for heightened clinical suspicion in endemic regions to prevent avoidable morbidity and inappropriate management [10–12].

## 2. Case Presentation

A 25‐year‐old man from rural Ethiopia presented to our hospital with crampy lower abdominal pain, recurrent hematochezia, progressive fatigue, and significant weight loss. He reported frequent exposure to freshwater. On examination, he appeared acutely ill with chronic features, including emaciation, pale conjunctiva, and tachycardia (pulse rate, 110 beats/minute; blood pressure, 110/75 mmHg). A digital rectal examination revealed multiple irregular, soft masses.

### 2.1. Differential Diagnosis

Several important differential diagnoses were considered in this case. Juvenile polyposis was initially suspected because of the presence of multiple rectal polyps. However, juvenile polyposis typically presents in childhood or adolescence with hamartomatous polyps. The histopathological examination of our patient revealed numerous *S. mansoni* ova surrounded by eosinophilic infiltrates, with no hamartomatous tissue. This finding effectively excluded the diagnosis of juvenile polyposis.

Inflammatory bowel disease (IBD), including ulcerative colitis and Crohn’s disease, was also considered because both conditions can present with rectal bleeding and polypoid lesions in the colon. However, colonoscopy did not reveal diffuse mucosal inflammation, skip lesions, or ulceration, which are characteristic of IBD. Furthermore, the histopathology lacked granulomas, chronic mucosal architectural distortion, or other features typical of IBD, making this diagnosis improbable.

Finally, colorectal cancer was a major concern, given the large pedunculated rectal polyp, friable surface, and contact bleeding, all of which mimic malignancy. However, biopsy specimens showed no dysplasia or malignant cells, and tumor marker levels were low. Instead, the presence of viable *S. mansoni* ova confirmed the *schistosomiasis* etiology. This underscores the importance of the histopathological examination in differentiating parasitic polyposis from neoplastic diseases, particularly in endemic areas.

### 2.2. Investigations

Initial hematological evaluation revealed severe microcytic hypochromic anemia with a hemoglobin level of 7.5 g/dL. The mean corpuscular volume (MCV) was reduced, and peripheral smear demonstrated hypochromic red cells with anisopoikilocytosis, consistent with iron deficiency anemia rather than chronic disease anemia. This was corroborated by low serum ferritin levels, reduced transferrin saturation, and mild hypoalbuminemia. Moderate peripheral eosinophilia was also observed, indicating a parasitic infection (Table 1).

**TABLE 1:** The initial and follow‐up hematological and biochemical results of the patient and the normal values.

Hematological and biochemical parameters	Initial presentation (12/1/2025)	3‐month follow‐up	Standard reference range	Clinical interpretation
Hemoglobin (Hgb)	7.5 g/dL	12.5 g/dL	13.5–17.5 g/dL (M) 12.0–15.5 g/dL (F)	Anemia

Mean corpuscular volume (MCV)	77 fL	88 fL	80–100 fL	Microcytosis

White blood cell (WBC) count	6 ∗ 10^3^/uL	5.8 ∗ 10^8^/uL	4.5–11.0 ∗ 10^3^/UL	Normal

Platelet (PLT) count	460 ∗ 10^3^/uL	320 ∗ 10^3^/uL	150–450 ∗ 10^3^/uL	Normal

Serum ferritin	15 ng/mL	Not done	30–300 ng/mL (M) 15–150 ng/mL (F)	Low/iron store depletion

Transferrin saturation	10%	Not done	20%–50%	Reduced/iron deficiency

Serum albumin	3.1 g/dL	Normal	3.5–5.0 g/dL	Mild hypoalbuminemia

Absolute eosinophil count (AEC)	1200/uL	300/uL	30–500/uL	Moderate initial eosinophilia

Stool wet mount microscopy with a high‐power field showed large yellowish‐brown oval eggs featuring a prominent, sharp lateral spine and a fully formed internal *miracidium*, confirming the presence of numerous *Schistosoma mansoni* ova with an active *schistosomiasis* infection. Abdominopelvic ultrasound revealed rectal wall irregularity and luminal narrowing, which initially raised concerns about malignancy. The tumor marker carcinoembryonic antigen (CEA) was 3 ng/mL (reference: 0–5 ng/mL). Further imaging with CT was not performed.

Colonoscopy revealed a large pedunculated rectal polyp measuring approximately 60 mm, located 8 cm from the anal verge (Figure 1). The polyp had a smooth but friable surface, with contact bleeding evident during manipulation and focal superficial ulceration. These features contributed to its malignant appearance and heightened the initial suspicion of colorectal cancer. In addition to the dominant lesion, multiple smaller sessile polyps were scattered throughout the rectal mucosa. These were less friable and showed no significant ulceration; however, their presence reinforced the impression of diffuse rectal polyposis.

**FIGURE 1:**
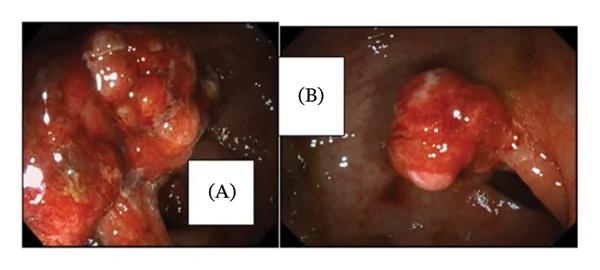
Colonoscopy findings at presentation: (A) large pedunculated rectal polyp measuring approximately 60 mm, located 8 cm from the anal verge, mimicking colorectal malignancy. (B) Multiple small sessile rectal polyps distributed along the rectal mucosa. Image credit: Firoomsis General Hospital, Jimma, Ethiopia.

The histopathological examination of the fully resected rectal polyp revealed numerous *S. mansoni* ova embedded in the lamina propria, with their characteristic hyperrefractive wall, oval shape, and lateral spine. Intense eosinophilic and chronic inflammatory infiltrates, consistent with active *Schistosoma* infection, were observed surrounding these lesions. The ova appeared viable, supporting ongoing parasitic activity rather than regressing or calcifying. Importantly, no granulomas, fibrosis, or calcified eggs were identified, which are features sometimes observed in chronic *schistosomiasis*. Similarly, there was no evidence of dysplasia or malignant transformation in the present study. The absence of neoplastic changes confirmed that the lesion was inflammatory and parasitic rather than neoplastic.

Taken together, the histopathological findings established *schistosomal* polyposis as the definitive diagnosis, ruling out juvenile polyposis, IBD, and colorectal cancer as differential diagnoses. The correlation between endoscopic appearance and pathology underscores the importance of biopsy in differentiating parasitic polyps from malignant or inflammatory conditions in endemic regions (Figure 2).

**FIGURE 2:**
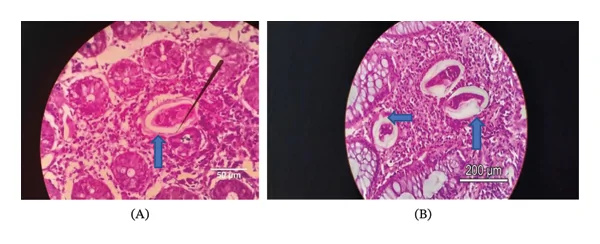
Histopathological findings of colonic tissue samples stained with eosin and hematoxylin: (A) A 40 × magnification showing numerous *Schistosoma mansoni* ova embedded in the lamina propria of the rectal mucosa (blue arrow). (B) 10 × magnification showing intense eosinophilic and chronic inflammatory infiltrates surrounding the *Schistosoma mansoni* ova with their characteristic hyperrefractive wall, oval shape, and lateral spine (blue arrows) without evidence of dysplasia or malignancy. Image credit: Firoomsis General Hospital, Jimma, Ethiopia.

### 2.3. Management and Follow‐Up

The patient was treated with praziquantel at a total dose of 40 mg/kg, administered in two divided doses, which is the standard regimen for *S. mansoni* infection. This ensured the effective eradication of parasites. Supportive therapy included daily oral iron supplementation with ferrous sulfate to correct iron‐deficiency anemia. Nutritional counseling was also provided to address weight loss and improve dietary iron intake.

Although the patient presented with severe anemia (hemoglobin, 7.5 g/dL), blood transfusion was not performed. The decision was based on the patient’s hemodynamic stability, the absence of cardiovascular compromise, and expectation that iron supplementation would gradually restore hemoglobin levels. Close monitoring was undertaken, and the patient’s hemoglobin level improved to 12.5 g/dL within 3 months of therapy. Endoscopic management included complete resection of smaller polyps with a cold snare. For the largest polyp, we performed piecemeal resection with a hot snare. The electrocautery machine was an Erbe Vio 200 S monopolar unit with settings of effect 4 and coag 60. We did not use submucosal injection as the polyp was pedunculated. No postprocedure complications occurred; the polyp removal was complete, and no adjunctive hemostatic mechanisms were applied. Follow‐up colonoscopy at 3 months demonstrated complete healing at the polypectomy sites, normal rectal mucosa, and no recurrence, establishing short‐term resolution.

## 3. Discussion

*Schistosomiasis* continues to pose significant diagnostic and therapeutic challenges in endemic areas. Although intestinal *schistosomiasis* is well described, its manifestation as large rectal polyps mimicking malignancy remains underrecognized. In our patient, the presence of a 60‐mm pedunculated polyp with associated anemia and rectal bleeding created a clinical picture that was highly suggestive of colorectal carcinoma. This highlights the importance of maintaining a high index of suspicion for parasitic etiologies in endemic areas, especially when patients present with hematochezia, eosinophilia, and a history of exposure to freshwater [7–10].

Several case reports and series have documented *schistosomal* polyposis presenting as colorectal masses but most have described smaller lesions. The unusually large size of this case, coupled with multiple sessile polyps, underscores the potential for misdiagnosis and unnecessary radical surgeries. Comparative reports from Egypt and Nigeria have similarly emphasized that histopathological confirmation is essential before proceeding with colectomy or other invasive procedures [7, 8, 10]. Our findings reinforce this principle and demonstrate that endoscopic biopsy remains the cornerstone for differentiating parasitic polyposis from neoplastic diseases.

The clinical implications of these findings are considerable. First, clinicians must recognize that *schistosomal* polyposis is not a malignant process but rather an intense inflammatory reaction to deposited *schistosomes* [11, 12]. Second, appropriate therapy, such as praziquantel combined with endoscopic polypectomy, can yield rapid symptomatic improvement and hematologic recovery, as seen in our patient. Third, follow‐up colonoscopy is critical to confirm mucosal healing and exclude recurrence, supporting long‐term surveillance strategies in endemic regions.

From a public health perspective, the burden of *schistosomiasis* in Ethiopia remains high, with more than 18 million people living in endemic areas [5, 6]. This case illustrates how neglected tropical diseases can masquerade as malignancies, leading to costly and potentially harmful interventions if misdiagnosed. Strengthening diagnostic capacity, ensuring access to endoscopy and pathology, and integrating *schistosomiasis* screening into routine gastrointestinal evaluation are essential steps to reduce morbidity.

Finally, this case contributes to the growing literature on *schistosomal* colorectal polyposis by documenting a rare presentation of a giant 60‐mm rectal polyp in a young adult. The novelty lies not only in the polyp’s size but also in the clear demonstration of the clinicopathologic correlation and favorable outcomes with conservative management. Early recognition and treatment can prevent complications, avoid surgery, and improve patient outcomes in resource‐limited settings. While there are no established protocols for monitoring rectal polyposis caused by *Schistosoma*, case studies and literature reviews suggest that parasitologic reevaluation should be done within 1–3 months after treatment. Endoscopic monitoring is determined by the initial size of the polyps and ongoing symptoms, and laboratory tests are conducted to check for anemia and eosinophilia. Consequently, our patient follow‐up mirrored the approaches in these studies. Additionally, because chronic stages of the disease result in low egg‐shedding rates, extended laboratory observation is necessary [7, 13]. Addressing the persistent public health issue of *schistosomiasis* requires providing communities with access to clean and safe water, maintaining public education on the proper use of fresh water, and seeking early treatment upon the appearance of symptoms and signs of *schistosomiasis*.

## 4. Limitation

The relatively short‐term follow‐up and single‐case format may limit the generalizability of the findings. Despite the initial suspicion of malignancy, computed tomography (CT) was not conducted due to resource constraints. The diagnosis was instead established through colonoscopy, histopathology, and a normal CEA level. Subsequent patient follow‐up demonstrated resolution of symptoms, signs, and initial colonoscopy findings, thereby negating the absolute necessity for CT scans.

## 5. Conclusion

In areas where *schistosomiasis* is prevalent, rectal polyps caused by the disease can be easily mistaken for colorectal cancer, making it crucial to confirm the diagnosis through histopathological and parasitological methods to avoid unnecessary radical surgery. Effective treatment involves a combination of endoscopic polypectomy, praziquantel medication, and supportive care, which leads to positive results, such as resolution of the polyp and associated complications. Maintaining long‐term control over the disease requires repeated colonoscopy checks. At the same time, broader efforts, such as increasing awareness, improving sanitation, and implementing screening programs, are vital to lessen the future impact of gastrointestinal *schistosomiasis*.

**Nomenclature**
Cm: Centimeter(s)
CT: Computed tomography
F: Female
fL: Femtoliter(s)
g/dL: Grams per deciliter
Hgb: Hemoglobin
IBD: Inflammatory bowel disease
M: Male
MCV: Mean corpuscular volume
mg/kg: Milligrams per kilogram
mm: Millimeter(s)
mmHg: Millimeters of mercury
ng/mL: Nanograms per milliliter
S.: Schistosoma (genus name in *S. mansoni*, *S. haematobium*, *S. japonicum*, and *S. mekongi*)
uL: Microliter(s)
μm: Micrometer

## Author Contributions

Alemayehu Abebe Guji, Birhanu Gudeta Geleta, and Melese Abere Gizaw contributed to the conception, design, investigation, analysis, and drafting of the initial manuscript. Mohammed Merga Ayana, Kedir Negesso Tukeni, and Gashahun Mekonnen Dissassa participated in supervision, data analysis, editing, and curation.

## Funding

No funding was received for the conduct of this study or preparation of the manuscript.

## Disclosure

All authors reviewed, modified, and approved the final version of the manuscript.

## Ethics Statement

This case report was not subject to formal ethical approval. Patient confidentiality was strictly maintained, and all data were used solely for this publication without any identifying information.

## Consent

Written informed consent was obtained from the patient for the use of clinical data and images in this study. All personal identifiers were excluded from the study. A copy of the written consent is available for review by the editor‐in‐chief of this journal.

## Conflicts of Interest

The authors declare no conflicts of interest.

## Data Availability

The data that support the findings of this study are available from the corresponding author upon reasonable request.

## References

[1] Ponzo E. , Midiri A. , Manno A. , Pastorello M. , Biondo C. , and Mancuso G. , Insights into the Epidemiology, Pathogenesis, and Differential Diagnosis of Schistosomiasis, European Journal of Microbiology and Immunology. (May 2024) 14, no. 2, 86–96, 10.1556/1886.2024.00013. PMC11097794 38498078

[2] Colley D. G. , Bustinduy A. L. , Secor W. E. , and King C. H. , Human Schistosomiasis, The Lancet. (June 2014) 383, no. 9936, 2253–2264, 10.1016/s0140-6736(13)61949-2. PMC4672382 24698483

[3] Costain A. H. , MacDonald A. S. , and Smits H. H. , Schistosome Egg Migration: Mechanisms, Pathogenesis and Host Immune Responses, Frontiers in Immunology. (December 2018) 9, 10.3389/fimmu.2018.03042. PMC6306409 30619372

[4] Nelwan M. L. , Schistosomiasis: Life Cycle, Diagnosis, and Control, Current Therapeutic Research. (January 2019) 91, 5–9, 10.1016/j.curtheres.2019.06.001. PMC6658823 31372189

[5] Ponpetch K. , Erko B. , Bekana T. et al., Environmental Drivers and Potential Distribution of *Schistosoma mansoni* Endemic Areas in Ethiopia, Microorganisms. (October 2021) 9, no. 10, 10.3390/microorganisms9102144. PMC8541272 34683465

[6] Magero V. O. , Kisara S. , Suleman M. A. , and Wade C. M. , Distribution of the Schistosome Intermediate Snail Host *Biomphalaria pfeifferi* in East Africa’s River Systems and the Prevalence of *Schistosoma mansoni* Infection, Transactions of the Royal Society of Tropical Medicine and Hygiene. (2025) 119, no. 3, 253–265, 10.1093/trstmh/trae115. PMC11887620 39656884

[7] Emara M. H. , Mahros A. M. , Rasheda A. M. et al., Schistosomal (Bilharzial) Polyps: Travel Through the Colon and Beyond, World Journal of Gastroenterology. (July 2023) 29, no. 26, 4156–4165, 10.3748/wjg.v29.i26.4156. PMC10354569 37475844

[8] Ahmed M. H. , Emara M. H. , Elfert A. A. et al., Persistent Colonic Schistosomiasis Among Symptomatic Rural Inhabitants in the Egyptian Nile Delta, Mediterranean journal of hematology and infectious diseases. (May 2021) 13, no. 1, 10.4084/mjhid.2021.033. PMC8114893 34007421

[9] Orare K. , Mohamed A. , Rajul A. , and Gatheru J. , Colonic Polyps: A Rare Clinical Manifestation of Schistosomiasis (A Case Report), Pan African Medical Journal. (2022) 42, no. 1. 10.11604/pamj.2022.42.187.34227 PMC9508373 36212935

[10] Mulate S. T. , Nur A. M. , Tasamma A. T. et al., Colonic Schistosomiasis Mimicking Cancer, Polyp, and Inflammatory Bowel Disease: Five Case Reports and Review of Literature, World Journal of Gastrointestinal Endoscopy. (2024) 16, no. 8, 472–482, 10.4253/wjge.v16.i8.472. PMC11325876 39155995

[11] Verjee M. A. , Schistosomiasis: Still a Cause of Significant Morbidity and Mortality, Research and Reports in Tropical Medicine. (December 2019) 31, 153–163, 10.2147/rrtm.s204345. PMC6997417 32099508

[12] Al-Zubaidi A. M. , Bashanfer G. A. , Alqannas M. H. , and Al Atawi A. S. , Colonic Polyps an Unusual Manifestation of Schistosomiasis, The American Journal of Case Reports. (August 2020) 21, e923177–1, 10.12659/ajcr.923177. PMC7476750 32764533

[13] Koulali H. , Zazour A. , Khannoussi W. , Kharrasse G. , and Ismaili Z. , Colonic Schistosomiasis: A Case Report, World Journal of Gastrointestinal Endoscopy. (December 2022) 14, no. 12, 789–794, 10.4253/wjge.v14.i12.789. PMC9782564 36567820

